# Application of Visual Recognition Based on BP Neural Network in Architectural Design Optimization

**DOI:** 10.1155/2022/3351196

**Published:** 2022-09-30

**Authors:** Rui Liang, Po-Hsun Wang, Linhui Hu

**Affiliations:** ^1^School of Architecture and Urban Planning, Guangdong University of Technology, Guangzhou, Guangdong 510090, China; ^2^Faculty of Innovation and Design, City University of Macau, Taipa, Macau 999078, China; ^3^State Key Laboratory of Subtropical Building Science, South China University of Technology, Guangzhou, Guangdong 510090, China; ^4^College of Art and Design, Guangdong University of Technology, Guangzhou, Guangdong 510090, China

## Abstract

In order to establish the mapping relationship between architectural design parameters and building performance and optimize architectural design parameters, an architectural design optimization method based on BP neural network is proposed. The selected main design parameters of building ventilation include spacing coefficient, air outlet area, and height from the bottom of the window sill to the ground. Take the comprehensive performance of building ventilation design as the main optimization objective to optimize the building design. First, nine groups of samples of building optimization design are obtained through uniform experimental design. Then, based on the architectural design sample data obtained by BP neural network training, the mapping relationship between architectural design parameters and building performance is established, and based on this mapping, the optimal design parameters of the building are calculated. The research results have a certain reference value for architectural design optimization.

## 1. Introduction

In recent years, with the rapid development of China's basic industry, building structure design also needs to make continuous adjustments and changes with the development of the times [1–4]. At present, architectural design is the basic condition to ensure the safety and stability of the main body of the building structure [5]. Good structural design optimization can not only improve the appearance quality and functional value of the building but also help to improve the quality of people's daily lives [6].

However, the development of high quality has put forward higher requirements for architectural design [7, 8]. At present, most architectural designs pay too much attention to form and light function, and the current design situation of pursuing standardization and a lack of innovation conflicts with it. The architectural design needs optimization and innovation urgently [9, 10]. The main design problems include unreasonable apartment design, incomplete functional space, and insufficient community supporting facilities. Understand the important influence of design factors (such as house type and orientation) on life experience (such as ventilation).

According to statistics, among the many reasons for engineering quality problems, design-related accounts for 40%, ranking first [11–13]. It is precise because of the lack of refined design and design optimization that the defects in the design are not avoided or the landing effect is not good. The presentation effect is inconsistent with the publicity effect or causes quality defects, affecting normal use [14].

With the rapid development of computer technology, the whole society is haunted by machine learning, big data, and artificial intelligence [15, 16]. These technologies are constantly promoting the progress of human civilization and also affect everyone's studies and lives all the time. The goal of artificial intelligence is to understand the thinking mode of the human brain so that robots can learn and imitate this intelligent and unique way of processing information and can better serve human beings, improve productivity, and promote human progress. Deep learning is in full swing in the field of computers [17–19]. In recent years, it has also been a preliminary attempt to use deep learning to recognize and classify objects in the field of robots. However, because the deep learning model requires a large number of data samples, sometimes tens of thousands or even hundreds of thousands of sample data, the sorting of target recognition parts is limited by the difficulty of identifying object image collections [20–23]. In the complex building design environment, visual recognition based on BP neural network is one of the important means for building designers to obtain building environment information [24]. However, the current vision recognition technology based on BP neural network is far from reaching the intelligent level of the human vision system and cannot recognize and understand any building environment like human beings. Therefore, the application of visual recognition in building design optimization needs further research [25–27].

This study applies the visual recognition method based on the BP neural network to architectural design optimization. Firstly, the main problems in the application of the visual recognition method in architectural design are analyzed, and then, a visual recognition system based on the BP neural network is constructed. Finally, the system is applied to the actual architectural design optimization. The research method of this study has certain reference significance for architectural design optimization.

## 2. The Concept and Principle of Architectural Design

### 2.1. Architectural Design Concept

Zhan Kesheng, who first put forward the theory of optimization method in residential building design in China, theory of “optimal residential building design method” in 1984, divides residential building design into three systems, residential use function, building technical economy, and building modeling, and then parameterizes the design factors of each system, establishes a mathematical model, and carries out systematic mathematical evaluation [28, 29]. The optimal room unit and residential unit type are obtained in turn, up to the residential area. The proposed method is based on the social development reality at that time. It is a simple theoretical analysis and research. The optimization method adopts a more complex mathematical model, but it is difficult to match the rapid development of residential buildings in China.

Urban planning is to dynamically solve and coordinate the connection between various buildings and the overall image of the building complex, to continue the history of the city, and to look forward to the future of the city from an ecological and sustainable point of view. Urban planning and design are the premise of architecture and garden construction and provide conditions for the required space [30]. The progress of urban planning and design research also provides an unprecedented broad world for the development of architecture and gardening. The design of buildings reflects the characteristics and requirements of urban planning. Our country is constantly making progress and constantly advancing with the pace of the times, and strengthening architectural design strictly reflects the characteristics of urban planning. As we are constantly innovating, we should always follow the forefront of the times, pursue innovation and development, and make our buildings more powerful.

With the continuous progress of science, people's living standards are constantly improving, and people's pursuits are also constantly updated. China's urban planning will continue to change with the pace. With “the theory of a round sky and a round place” as a whole cultural concept, square has become the ideal model of human living space, which has laid the ideal form of China's square city from the deep-seated cultural awareness. Architectural design solves the coordination between individual building functions and the urban environment under the influence of urban regulatory detailed planning [30, 31]. These will be the characteristics of the city. Urban planning is the analysis and design of urban space, that is, the coordination of the regional space of human activities and the relationship between regional spaces [32]. In terms of breadth, it was previously believed that the three periods of point planning of industrial layout, linear development of transportation layout, and area planning with the goal of creating a better environment, and improving residential and working apartments did not solve the social disease of urban development [33]. We should explore a more holistic and holistic planning, that is, to establish an overall separation and development model of “nature space human system” within the territory.

In modern cities, residential buildings are mostly dense, and local wind fields are complex, making it easy to form local wind fields. The setting of architectural design parameters directly affects the wind pressure on the building surface, thus affecting the ventilation effect of the building room. How to reduce the negative impact of adverse wind fields on residents lives is a major challenge for architectural design optimization.

### 2.2. Design Principle

People-oriented is at the core of the scientific concept of development. It aims to achieve people's all-round development, pays attention to people's needs, and emphasizes full respect, understanding, and support for people [32]. Architecture is to provide services for people, so the principle of being people-oriented must be emphasized in architectural design, which should not only try to meet people's basic needs, including user needs, health needs, safety needs, etc., but also fully respect people's personalities and design in combination with people's personality characteristics.

Nowadays, with the increasingly serious problem of energy shortages, how to reduce energy consumption and save energy while developing the industry has become an important research topic [34, 35]. Some nonrenewable energy is extremely valuable. If human beings exploit it without restraint, sooner or later, they will face the day of energy exhaustion. Although some renewable energy sources are inexhaustible, they also need to incur a certain cost. Therefore, in the development of the construction industry, we must pursue low consumption and energy conservation, comprehensively use various energy-saving technologies and energy-saving materials in architectural design, maximize energy efficiency, reduce energy consumption, and reduce unnecessary energy waste [36].

The protection of the ecological environment and the construction of ecological civilization are the common responsibilities of all mankind. Only by building eco-friendly buildings, making them live in harmony with the ecological environment, becoming a part of the urban ecological composition, and avoiding damage to the ecological environment, can we conform to the scientific concept of development. Eco-friendly architectural design also lies in conforming to nature, respecting the objective laws of nature, and looking for ways to get close to nature. In other words, it is to combine architectural design with environmental planning [37].

No matter what type of architectural design, people should first meet the basic requirements of living comfort to ensure that the designed building is suitable for people to live. In real life, many factors will affect the comfort of buildings, such as air quality factors, temperature factors, humidity factors, light factors, and environmental factors. The common feature of these factors is that they all belong to the object of human perception. Therefore, in order to effectively create a comfortable and livable building, these factors should be fully considered in the design to ensure that people can obtain a comfortable living experience to the greatest extent.

## 3. Challenges Faced by Machine Learning in Related Fields

Although machine learning classification algorithms can deal with many complex classification problems, with the data becoming more complex and diverse, machine learning classification algorithm has encountered new challenges in learning objectives and classification efficiency.

### 3.1. Low Efficiency of Target Learning and Classification

Data in different application fields show high-dimensional characteristics. With the increase of redundant and irrelevant information in the data, the performance of machine learning classification algorithm decreases and the computational complexity increases. Machine learning classification algorithms generally need to use large samples to learn effectively. Big data does not mean that the number of training samples is sufficient. When the sample size is small and the features contain a large number of irrelevant features or noise features, the classification accuracy may be low and overfitting may occur.

**Figure 1:**
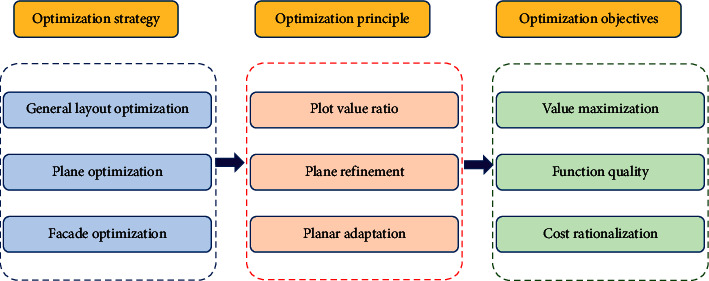
Correspondence diagram of optimized strategies, principles, and objectives.

Machine learning classification algorithms generally assume that the data set used for training is balanced, that is, the number of samples in each class is roughly equal, but in reality, the data are often unbalanced. Existing studies usually deal with imbalance and high-dimensional problems separately, but in practice, there are often data with the dual characteristics of imbalance and high-dimensional. At present, the strategies, principles, and objectives of machine learning optimization are shown in Figure 1.

In addition to the common binary classification problems, there are a lot of multiclassification problems in practical applications, especially the multiclassification problems of high-dimensional data, which pose challenges to the existing machine learning classification algorithms.

At present, data instances in the application of machine learning classification algorithms are represented by a large number of features. A good classification model depends on a feature set with high correlation. Removing irrelevant and redundant features can not only improve the accuracy of the model but also reduce the running time. Therefore, research on feature selection is becoming more and more important for the development of machine learning classification algorithms.

### 3.2. The Optimization Concept is Not Mature Enough

Integrating the green building design concept into the architectural design is an important content in the development of modern architecture. The beauty and use efficiency of buildings can be significantly improved through the green building design concept, and the energy conservation and consumption reduction and rational utilization of resources in the green building design can also have a good significance for environmental protection and have high application value. The infiltration of green building design concepts into architectural design needs to follow certain principles and strategies. According to the green building design concept, discussing the building site selection, application of environmental protection materials, and optimization of the housing structure, greening of landscape and other contents in modern architectural design can further optimize the architectural design and create a high-quality building project with ecological environmental protection significance and sustainable development.

The concept of green building design has a high application value in architectural design. When designing green buildings, we often need to consider the principles of environmental protection, efficiency, livability, and economy. The infiltration of green building design concept in current architectural design makes the design of building structure more reasonable and superior. Integrating green building design concept into building site selection design, environmental protection material selection, building structure design and green landscape design can better ensure the construction quality. The application of green building design concept in architectural design is gradually increasing. The integration of green building design concept can not only ensure the artistry and practicality of architectural design itself but also further improve the effect of building energy conservation and environmental protection, thereby promoting the development and progress of the construction industry.

### 3.3. Machine Learning Is Rarely Used in Architectural Design Optimization

**Figure 2:**
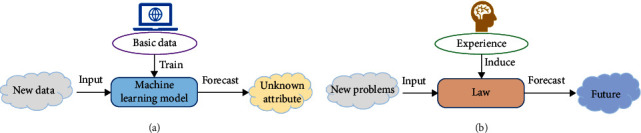
The difference between (a) machine learning and (b) human thinking.

With the development of computer technology and artificial intelligence, machine learning is becoming more and more widely used. Whether in military or civil fields, there are opportunities for machine learning algorithms. Machine learning is to automatically summarize the logic or rules behind things by selecting appropriate algorithms from data and predict the development of things according to the inductive results (models) and new data. Machine learning is to let the machine find the correlation in the data through the learning of a large amount of data [38–40]. In addition to relevance, human intelligence has no causal relationship with today's artificial intelligence. Machine learning does not understand causality. It only knows correlation, so this is the biggest difference between machine learning and human thinking, as shown in Figure 2.

Because the most difficult step of machine learning is to refine the problems in real production and life into machine learning problems, what this requires is researchers' deep insight into the actual problem itself. Moreover, no matter how accurate the prediction of the machine is, its result is worthless if it is not to answer human needs. Therefore, applying BP neural network to architectural design optimization and using machine learning methods to solve the problems existing in architectural design is a new research method, which is of great value for the development of architectural design optimization methods. However, when using the BP neural network for architectural design optimization, the most important thing is to build a machine learning problem. The optimization problem of architectural design is abstracted as a machine learning problem for subsequent neural network training and parameter optimization.

## 4. Construction of Architectural Design Optimization System Based on Machine Learning

**Figure 3:**
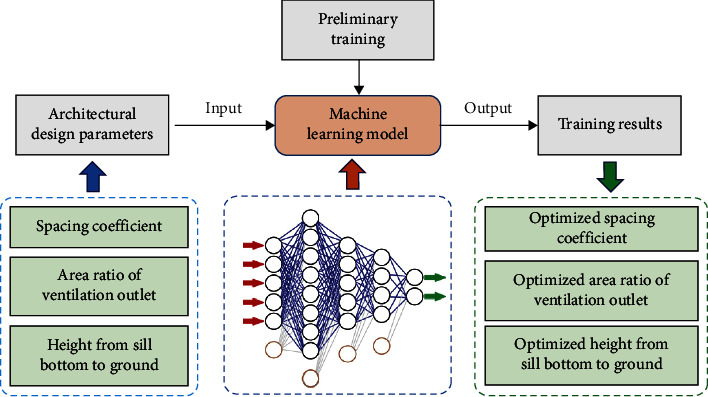
Optimization design of building ventilation.

In order to establish the mapping relationship between building design parameters and building ventilation performance and optimize building ventilation design parameters, a method based on uniform design and neural network is proposed. Take indoor ventilation as the optimization goal. In this study, the architectural design parameters that affect the natural ventilation effect of residential buildings are divided into two categories: outdoor design parameters and indoor design parameters. The outdoor design parameters are plane layout and building spacing, and each design parameter is designed with three change levels. The indoor design parameters are two influence parameters: ventilation outlet area and the distance from the outlet to the floor), and each design parameter is designed with three change levels. The sample data of optimal design are obtained through uniform test design method, and the mapping relationship between design parameters and bridge performance is established through BP neural network training sample data. Based on this mapping, the corresponding design parameters under the optimal performance state of the architecture are calculated, and finally, the parameter optimization is completed. The optimization design of building ventilation is shown in Figure 3.

### 4.1. Outdoor Parameters' Setting

**Figure 4:**
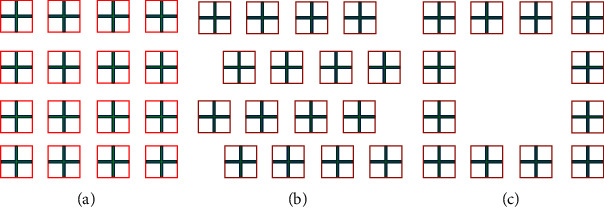
Three models of different building layouts: (a) parallel type; (b) staggered type; (c) enclosed type.

The most common community layouts of residential buildings are staggered, enclosed, and parallel. This paper mainly studies the impact of the three most common building layouts on the indoor natural ventilation effect, numbered A1, A2, and A3, respectively, as shown in Figure 4.

**Figure 5:**
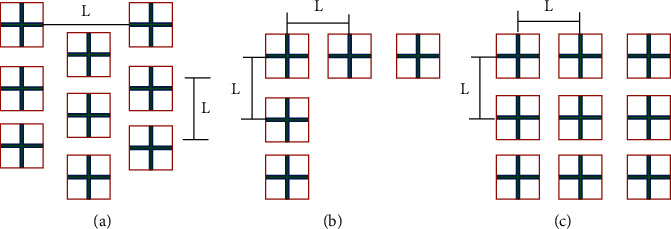
Schematic diagram of horizontal and vertical spacing of buildings.

The design of building spacing is one of the important factors to be considered in the plane design of buildings. The building spacing should meet the relevant provisions of ventilation, fire prevention, sunshine, daylighting, noise prevention, and so on. In this study, the influence of building spacing on natural ventilation is characterized by the spacing coefficient in the building group. The spacing coefficient represents the ratio of building spacing to the height of the main building (the highest building height), as shown in the following formula:

(1) $$\begin{array}{c} \Psi =\frac{L}{H}\text{,} \end{array}$$

where *ψ* is a dimensionless variable, *H* is the height of the main building, *m*, and *L* is the distance between buildings, *m*. The horizontal and vertical spacing of buildings with different plane layouts is shown in Figure 5.

Ratio of the openable area of naturally ventilated windows to the area of ventilated rooms *σ*. To characterize the size of the ventilation outlet area, set three levels of change *σ* = 8%, *σ* = 10%, and *σ* = 12%, numbered F1, F2, and F3 respectively. And set two window openings in the natural ventilation room, and the net height of the window opening is equal, which is 1.5 m. The size is achieved by changing the opening width and the size setting of different ventilation outlet areas.

**Figure 6:**
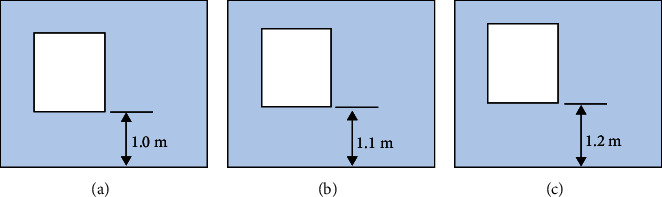
Schematic diagram of distance between air outlet and ground.

Studies have shown that the optimal design of the distance from the air outlet to the ground can change the wind field characteristics of natural ventilation rooms. In daily residential buildings, the height from the bottom of the window sill to the ground is generally set in the range of 0.9–1.2 m. In this study, the height from the bottom of the window to the floor is used to characterize the influence of the distance from the air outlet to the ground (floor) on the natural ventilation effect. The height from the bottom of the window that can be naturally ventilated to the floor is set to be 0.9 m, 1.1 m, and 1.2 m, numbered H1, H2, and H3, respectively, as shown in Figure 6.

### 4.2. Architectural Design Optimization System Based on BP Neural Network

#### 4.2.1. Principle of BP Neural Network

**Figure 7:**
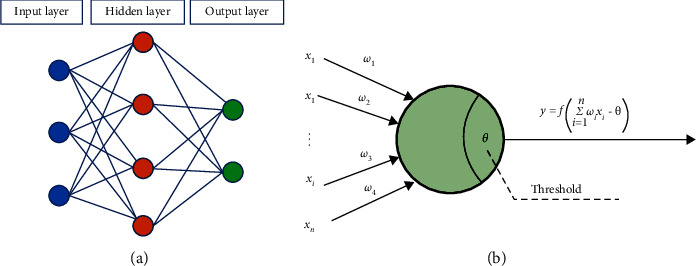
Schematic diagram of typical neural network structure. (a) BP neural network model. (b) Neuron topology.

As one of the ways to realize machine learning, neural network is a computer deep learning technology that simulates human brain neural networks. The BP neural network is a typical three-layer neural network. Theoretically, it can approach any continuous function infinitely. In other words, through the training of a certain number of samples, a BP neural network can obtain the linear and nonlinear relationship between some attributes or features. As shown in Figure 7, a typical BP neural network structure mainly includes neurons (input, hidden, and output layers) and connecting lines. The connecting line between neurons represents the weighted transmission of values in a specific direction.

#### 4.2.2. Optimization Objectives

In general, the inevitable consideration in the optimization of building ventilation design is the requirements of ventilation. At the same time, the economy should be fully considered while meeting the ventilation requirements. Therefore, the optimization objective should include the requirements of strength, deflection, and the amount of building materials (representing the economic level). This study takes the strength and deflection of the mid-span section and the concrete dosage of the whole bridge as the optimization objectives. Obviously, this is a multiobjective optimization problem. The calculation of the multiobjective optimization problem is cumbersome, and it is difficult to establish an accurate mathematical model. Therefore, it is considered to use the formula scoring method to convert the multiobjective optimization problem into a single objective optimization problem. Three optimization goals into one optimization goal are as follows:

(2) $$\begin{array}{c} K=\frac{\Psi }{\varphi }+\frac{S}{s}+\frac{H}{h}\text{,} \end{array}$$

where *K* is the design comprehensive performance for building ventilation, Ψ is spacing coefficient adopted in design, 0.5–1.0, Φ is the lower limit of spacing coefficient, 0.5, *S* is the area ratio of ventilation outlet, range is 9%–11%, *s* is the lower limit of area ratio of ventilation outlet, 9%, *H* is height from sill bottom to ground, m, range is 1–1.2 m, and *h* is the lower limit of sill bottom height from the ground, m.

Then, according to formula (2), calculate the maximum building spacing coefficient, ventilation outlet area, and the height from the bottom of the windowsill to the ground corresponding to a group of building optimization design parameters under extreme conditions, and then, calculate the comprehensive performance of the building design under the building design parameters.

#### 4.2.3. Optimization Calculation of Architectural Design

On the premise of clarifying the optimization objectives and main structural design parameters of building ventilation design, it is necessary to establish a mathematical model between structural design parameters and building performance in order to solve the building design parameters under optimal performance. However, it is usually impossible to establish the explicit functional relationship between building performance and design parameters. Therefore, the BP neural network is considered to establish the mapping relationship between building design parameters and building performance, so as to complete the optimization according to the mapping relationship.

The key of the BP neural network lies in the selection of training samples. The training samples need to be as representative as possible. It is best to evenly distribute the value space of the whole function. Uniform experimental design is an experimental design method based on this demand, so this study considers adopting the uniform experimental method to calculate the initial value of the model. The test factors of this study are three parameters, that is, the test has three factors, and the level number should be three times the number of factors. The comprehensive performance of building ventilation design corresponding to the test design table and each parameter combination is shown in Table 1. The data in Table 1 can be used as the sample data of BP neural network training.

#### 4.2.4. Optimization of Architectural Design Parameters

The BP neural network can imitate the interconnection between neurons in the human brain and the processing method of information and establish the nonlinear mapping relationship between input data and output data by learning the selected training data samples. Usually, the three-layer BP neural network can solve most mapping problems. Therefore, the neural network of this study adopts a three-layer neural network.

Due to the different dimensions of the parameters in this study, there are great differences in values. In order to avoid the small value information being flooded by the large value, all data samples need to be normalized according to

(3) $$\begin{array}{c} Y=0.8\frac{X-X_{\min}}{X_{\max}-X_{\min}}+0.1\text{,} \end{array}$$

where *Y* and *X* are the values of each group of parameters after normalization and before normalization, respectively, and *X*_max_ and *X*_min_ are the maximum and minimum values of each group of parameters, respectively. After normalization, the input and output data are all within the range of [0.1, 0.9], which can not only retain the relative information of the original data but also speed up the network learning speed and improve the network convergence ability.

In this study, an empirical formula (3) is used to approximate the number of neurons in the hidden layer:

(4) $$\begin{array}{c} p=\sqrt{m+n+a}\text{,} \end{array}$$

where *p* is the number of neurons in the hidden layer, *m* is the number of input layer units, *n* is the number of output layer units, and *a* is a positive integer between [1, 10].

**Figure 8:**
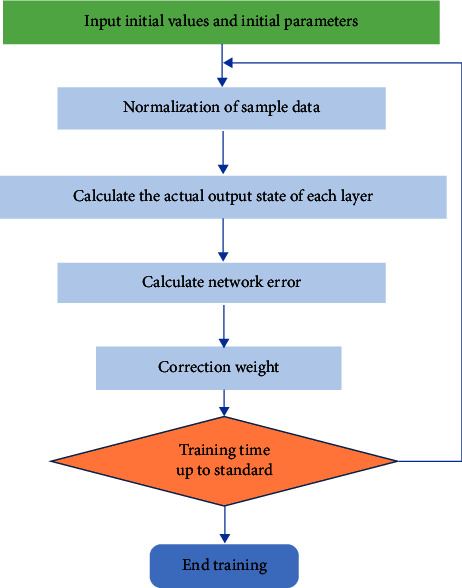
Optimal parameter solving process.

In the calculation process, the number of training is 30. The BP neural network established in this study is used to process the sample data in Table 1, normalize the sample data in Table 1 according to formula (3), and then process it with the BP neural network model. The process is shown in Figure 8.

According to the training results of the neural network, the spacing coefficient used in the design optimization of building ventilation design is 0.88, the area ratio of ventilation outlets is 10.02%, the height from the bottom of the window sill to the ground is 1.1 m, and the value of K is 1.521.

## 5. Discussion

The research for this study is a small part of the related research in the ventilation optimization of architectural design, and the existing machine learning is applied to the design optimization. The research for this paper involves three kinds of architectural design parameters. Based on the idea of this research, we can further consider the impact of more architectural design parameters on the natural ventilation effect in the future, such as building density, building height, and ventilation outlet shape.

The design spacing coefficient for building ventilation is 0.88, the area ratio of ventilation outlets is 10.02%, and the height from the bottom of the window sill to the ground is 1.1 m.

In addition, the concept of green building can also be taken into account in the model of this study. The concept of green building design has high application value in architectural design. When designing green buildings, we often need to consider the principles of environmental protection, efficiency, livability, and economy. The infiltration of green building design concept into current architectural design makes the design of building structure more reasonable and superior. Integrating green building design concept into building site selection design, environmental protection material selection, building structure design, and green landscape design can better ensure the construction quality. The application of green building design concept in architectural design is gradually increasing. The integration of green building design concept can not only ensure the artistry and practicality of architectural design itself but also further improve the effect of building energy conservation and environmental protection, thereby promoting the development and progress of the construction industry. The research methods and means of the study can provide data support for scholars at home and abroad in theory and methods.

## 6. Conclusions

Based on the analysis of the main principles and characteristics of current architectural design optimization, this study selects the optimization objectives and parameters of architectural design. Aiming at the optimization of building ventilation design, the building design optimization is carried out based on the method of the BP neural network. The main parameters of architectural design optimization are obtained through calculation. The main conclusions are as follows:

1. The optimization objective of architectural structural design parameters is determined, and three main structural design parameters that have a significant impact on architectural design optimization are summarized.
2. The sample data for building design parameter optimization are obtained through the experiment. Then, a BP neural network is used to establish the mapping relationship between building design parameters and building ventilation performance. Finally, based on the existing data and mapping relationship, the optimal structural design parameters of the bridge are calculated.
3. According to the BP neural network training results, the optimal design parameters used in building ventilation design optimization are as follows: the spacing coefficient is 0.88, the ventilation outlet area ratio is 10.02%, and the height from the bottom of the windowsill to the ground is 1.1 m.

## Figures and Tables

**Table 1:** Experimental results.

Number	Ψ	*S* (%)	*H* (m)	*K*
1	0.53	9.1	1.1	1.044
2	0.74	9.8	1.05	1.742
3	0.85	10.2	1.15	1.573
4	0.91	9.6	1.09	1.683
5	0.69	10.8	1.18	1.358
6	0.78	10.1	1.05	1.328
7	0.56	9.4	1.12	1.552
8	0.95	9.9	1.08	1.984
9	0.87	10.9	1.15	1.142

## Data Availability

The dataset used in this paper can be obtained from the corresponding author upon request.
